# Cytoplasm-localized SIRT1 downregulation attenuates apoptosis and cell cycle arrest in cisplatin-resistant lung cancer A549 cells

**DOI:** 10.7150/jca.44383

**Published:** 2020-05-18

**Authors:** Hyeran Yu, Young Mee Kim, Moonjae Cho

**Affiliations:** 1 Department of Biochemistry School of Medicine, Jeju National University, Jeju, Korea; 2 Institutes of Medical Science, Jeju National University, Jeju, Korea

**Keywords:** Cisplatin-resistant lung cancer, EMT, SIRT1, Cell cycle arrest, Apoptosis

## Abstract

**Objective**: We propose that sirtuin (SIRT) may induce a pro-apoptotic effect by deacetylating transcription factors in A549 cells: depletion of sirtuin-1 (SIRT1) induced cell cycle arrest in cisplatin-resistant A549 (A549/CADD) cells.

**Methods**: Protein and mRNA levels of SIRT1 were investigated using western blot and RT-PCR. In A549 and A549/CADD cells, the cytotoxicity of cisplatin administration was evaluated by MTT assay, proliferation was measured by ECIS, and the cell cycle distribution was analyzed using FACS. Cells were transfected with pcDNA3.1-Myc-SIRT1 or pcDNA3.1-Myc-Control vectors to analyze the impact of SIRT-1 on cisplatin induced drug resistance. SIRT1 localization was studied using immunofluorescence analysis. In addition, immunoprecipitation and 20S proteasome activity assay were performed to examine the relationship of SIRT1 with the proteasome complex.

**Results**: A549/CADD cells exhibited a mesenchymal-like cell characteristic. SIRT1 expression was markedly decreased in A549/CADD cells. We observed that cisplatin regulates p53 stability through the depletion of ubiquitination following SIRT1 downregulation. Furthermore, cisplatin treatment increased proteasomal activity and significantly decreased cytoplasmic SIRT1 protein levels in A549/CADD cells.

**Conclusion**: In this study, we found SIRT1 to be depleted in A549/CADD cells and also determined the underlying resistance mechanism which may act as novel therapeutic targets in overcoming drug resistance.

## Introduction

With more than 1.7 million deaths in 2018, lung cancer has become the most common cause of cancer-related death worldwide. Among cancer deaths faced by the Americans in 2018, nearly 25% were due to lung cancer. Lung cancer is often classified into small cell lung cancer (SCLC) and non-small cell lung cancer (NSCLC) with 87 of every 100 lung cancer cases are diagnosed as NSCLC. Treatment strategies towards lung cancer malignancy are often associated with pernicious side effects. Understanding the molecular mechanisms underlying its progression at different biological levels, such as genetic, epigenetic and translational levels, has great influence in terms of diagnosis, prognosis and treatment outcome. Among several chemotherapeutic drugs employed in the treatment of lung cancer, platinum-based chemotherapeutic drugs (cisplatin, paraplatin, oxaliplatin) are considered as the standard first-line therapy, and are commonly used. Although the initial outcome is promising, later stages of the treatment program are not, as the cancer cells acquire drug resistance through the underlying metabolic programs. Cisplatin (cis-diamminedichloroplatinum (II), CDDP) exerts its anti-cancer action via interacting with DNA thereby imposing DNA damage and apoptosis. 1 Apoptosis results from the activation of apoptotic signals like caspase-3, poly ADP-ribose polymerase (PARP), and Bax. Molecular defects in the apoptotic pathway induces chemoresistance in these cancer cell types, resulting in the development of inherent and acquired resistance to such drugs, thereby limiting their application. Further, prolonged cisplatin treatment leads to chemoresistance via genetic or epigenetic changes with unclear underlying mechanisms.

The mammalian sirtuin (SIRT) protein family comprises seven members, which differ in their subcellular localization and function. Among the seven members, the role of sirtuin 1 (SIRT1), and NAD^+^-dependent deacetylase involved in several processes such as cell death, senescence, stress response, and cancer, is well documented 2. The most widely known substrates of SIRT1 is p53, a tumor suppressor with a critical role in cell-cycle regulation and apoptosis 3. SIRT1 inhibits p53 transcriptional activation by deacetylating p53, following DNA damage through chemotherapy. NADPH oxidases also regulate cell-cycle entry via the p53-dependent pathway 4. In cancer, SIRT1 might play a promotional or suppressive role, depending on the cancer type and organs involved. In previous lung cancer studies, SIRT1 has been shown to have oncogenic properties. Overexpression of SIRT1 is associated with tumorigenesis and apoptosis. SIRT1 might facilitate chemoresistance in cancer cells through regulating the adaptive response to chemotherapy-induced stress 5. However, there are studies indicating the role of SIRT1 as a tumor suppressor. Nevertheless, SIRT1 has been shown to regulate epithelial-mesenchymal transition (EMT) in cancer cells 6. The role of SIRT1 in carcinogenesis is controversial and remains unclear. In a recent study, low SIRT1 expression levels were associated with poor prognosis in lung cancer patients 7. In this study, we propose to explore the association between downregulated SIRT1 expression and cisplatin resistance in NSCLC.

EMT is implicated in cancer progression and is thought to influence cancer treatment (8). Until now, many reports have demonstrated that EMT is involved in drug resistance in NSCLC 8. EMT leads to a loss of epithelial characteristics, including cell-cell junctions, and polarity, and a gain of mesenchymal properties. Sirtuin, an NAD^+^ dependent acetylase, regulates many pathophysiological conditions, including cancer 2. Compared to other histone deacetylases, SIRT1 deacetylates many proteins, p53 being one of the main targets 9. The dual functions of SIRT1 in cancer progression introduce many questions regarding its function in the regulation of tumor progression, particularly tumor suppression. For example, in HMLER breast cancer cells, reduced SIRT1 levels are associated with increased metastases 10, whilst high SIRT1 expression promoted tumorigenesis and is associated with poor prognosis in colorectal carcinoma patients 11. Furthermore, the nucleoplasmic shuttling of SIRT1 12 mediated anticancer activity, its role in the deacetylation process of p53, and SIRT1-mediated regulation of the cisplatin-resistant cell cycle mechanism, are largely unexplored.

CDDP has become the prop of anticancer treatment over the past 20 years. Thus, it became indeed necessary to clarify the mechanisms that participate in CDDP resistance cancer progression. Though initial focus was directed on internal characteristics such as drug transporters, detoxification enzymes, DNA damage repair systems etc., which are the key events in sensitizing the cancer cells towards this platinum-based chemotherapy treatments, yet recently researches targeting the underlying chemo-resistive signaling pathways is gaining more interest. In this current article, we focused on the relationship between cisplatin resistance and SIRT1 protein expression in cisplatin resistance non-small cell lung cancer cells (A549).

## Materials and Methods

### Cell culture

The A549 and A549/CADD cells were produced by culturing the cells in Dulbecco's modified Eagle's medium (Gibco, Wellesley Hills, MA, USA), supplemented with 10 % Fetal bovine serum (Omega, Tarzana, CA, USA) and 1% penicillin/streptomycin. Cells were incubated at 37 °C in 5 % CO_2._ We repeatedly subculture A549 cells in the presence of increasing concentration (0.1, 0.2, 0.4 and 0.5 mg/ml) of cisplatin over 6 months. Thereafter, we transfer the cisplatin resistance A549 cells (A549/CADD) through colony selection (micropipette tip) to fresh plates and maintained with 2mg/L cisplatin over 2 months. Before the start of each experiment described in this article, we treated the A549/CADD cells with appropriate concentration of cisplatin (as mentioned) for 1 week.

### Reagents and antibodies

Monoclonal antibodies against human SIRT1, p53, 20S Proteasome β1, Mox1, gp91-phox, cyclin D1, N-cadherin and β-actin were purchased from Santa Cruz Biotechnology, Inc. (Santa Cruz, CA, USA), and antibodies against vimentin, ubiquitin, acetyl-p53(Lys382), phospho-Rb(Ser807/811), Caspase-3, p21 Waf1/Cip1, Bcl-2, PARP, Akt, Bax and GAPDH were from Cell Signaling Technology (Danvers, MA, USA). The anti-E-cadherin antibody was purchased from BD Transduction Laboratories (Franklin Lakes, NJ, USA), and MG-132 (Carbobenzoxy-L-leucyl-L-leucyl-L-leucinal) was purchased from Calbiochem (Burlington, MA, USA). The anti-Nox4 antibody was purchased from Novus Biologicals (Littleton, CO, USA). The antibodies against proteasome subunit β type-2 were purchased from Abcam (Cambridge, UK), and proteasome subunit β type-5 was purchased from Proteintech (Rosemont, IL, USA). The proteasome 20S Activity Assay Kit and cisplatin were purchased from Sigma-Aldrich (St. Louis, MO, USA). Anti-mouse, anti-rabbit secondary antibodies were purchased from Santa Cruz Biotechnology, Inc. The primary antibody was diluted with 5% skimmed milk in 0.1%Tween20-TrisHCl-buffered saline (TBST) in the concentration range of 1:1000 to 1:2000; while the secondary antibodies were diluted in TBST solution in the concentration range from 1:10000 to 1:20000.

### Western blot analysis

After appropriate treatment, cells were washed two times with PBS and lysed in RIPA buffer for 20 min in a 4 °C shaker. The lysates were collected by scraper and centrifuged at 14,000 RPM for 15 min. The protein concentrations of the cell extract were determined using a BCA Protein Assay Kit (Thermo Scientific, Rockford, IL, USA). Equal amounts of each protein samples (~30μg/lane) were separated by 10 or 15% SDS-polyacrylamide gel electrophoresis, and transferred onto Amersham Protran 0.45 NC nitrocellulose membranes (GE Healthcare Life Science, Seoul, Korea), and blocked with 5% milk in TrisHCl-buffered saline (TBS) containing 0.1% Tween 20 (TBST) for 1 h. Thereafter, the membranes were incubated at 4 °C overnight with various primary antibodies. After washing with TBST, the membranes were incubated with mouse and rabbit HRP-conjugated secondary antibodies at 23 °C and developed using ECL solution (LPS Solution, Daejeon, Korea), a chemiluminiscent detection method. The band intensities were quantified using Image J software (NIH, Bethesda, MD, USA) to analyze the difference in expression relative to the control.

### RNA extraction, reverse transcription

Total RNA was extracted from the cultured cells using Trizol reagent (Invitrogen, Carlsbad, CA, USA). The cDNA from total RNA was synthesized using a reverse transcriptase kit (Promega, Madison, WI, USA). PCR primers for human SIRT1 were as follows: SIRT1 forward (5'-AGCTACAGGATCCACGGGTC-3'), SIRT1 reverse (5'-CATGAAGAGGTGTGGGTGGC-3'). After the polymerase chain reaction, the PCR products were separated on a 1% agarose gel containing 0.002% nucleic acid staining solution (RedSafe, iNtRON Biotechnology Inc.). The band intensities were quantified using Image J software to analyze the difference in expression relative to the control.

### Cell viability assay

Next, 200 μL of the cell suspension (1.5 × 10^4^ cells/mL) was seeded in 96-well plates. Cells were treated with cisplatin or MG132 for 24 or 72 h. Thereafter, 10 µL MTT solution (10 mg/mL; Sigma-Aldrich) was added to each well, and cells were incubated for 4 h at 37 °C. Then, the medium was gently removed via suction tip and replaced with 150 μL DMSO, and the plate was incubated for 30 min at 23 °C with shaking. Sample absorbance was measured at 570 nm in a spectrophotometer (Tecan Sunrise, Tecan, Männedorf, Switzerland).

### Cell proliferation analysis

Electric Cell-substrate Impedance Sensing (ECIS) is a experimental method used to analyze cell proliferation or migration in real time, and the resistance value is shown as a graph. As cells grow, resistance detected by metal is increased and it invert to impedance through self-program. When cells are added to the ECIS arrays and attach to the electrodes, they act as insulator increasing the impedance. Therefore, as cells grow and cover the electrodes, the current is impeded in a manner related to cells covering the electrode. Electrode-stabilizing solution (200 μL) containing 10 mM L-cysteine was added to each well and kept at 23 °C for 10 min; the plate was washed with DMEM, and the cell suspension (3 × 10^3^ cells/mL) was seeded in 6-well plates. After 24 h, the medium was gently removed, and the cells were treated with 100 μL DMEM containing cisplatin and MG132 and incubated at 37 °C. Using the program, we transferred the excel data sheet and calculated normalized impedance.

### Cell cycle analysis

Cells (6 × 10^4^ cells/mL) were treated with cisplatin for 24 h or 1week. Next, the cells were washed with PBS, the supernatant was removed, and the cell pellets were incubated with 70% ethanol. Thereafter, the cell pellets were centrifuged and re-suspended in PI mixture buffer (50 μL of PBS, 100 μg/ml PI, and 10 μL RNase) at 37 °C for 30min. Finally, a minimum of 10,000 cells/sample were collected, and the samples were analyzed by flow cytometry (BD FACSCalibur, BD Biosciences, Franklin Lakes, NJ, USA).

### Cell transfection

The pcDNA3.1-Myc-SIRT1 and pcDNA3.1-Myc-Control vectors were provided by Dr. Han-Geuk Seo **(**Department of Animal Biotechnology, Konkuk University, Seoul, Korea). The cDNA encoding human SIRT1 (GenBank accession no. AF083106) was cloned into the pcDNA3.1/Myc vector. The cell suspension (5 × 10^4^ cells/mL) was seeded in 6-well plates. Cells were transfected with 2.5 μg pcDNA3.1-Myc-SIRT1 or pcDNA3.1-Myc-Control vector using transfection reagent (Omics Bio, LaiChiKok, Hong Kong). To knock down SIRT1 expression, cells were transfected with 100 nM of control or SIRT1-specific small interfering RNAs (Bioneer, Daejeon, Korea) using transfection reagent in serum-free medium. For this, RNAs and transfection reagent mix were vortexed with serum-free medium in separate tubes, and were allowed to settle for 5min. Then, both the RNA and transfection reagent solution were mixed and vortexed again, and made to settle for 15 min. This mixture was added to the serum-free medium in the cell culture plate containing cells ready to be transfected. Thereafter, the plate was incubated at 37 °C, and was shaken once every 30 min for 4 h to obtain transfected cells. After 24 h, cells were collected for western blotting and proteasome activity assay.

### Protein stability assay

Cells were simultaneously treated with 50 µg/mL cycloheximide (CHX) for 6 h. Subsequently, cells were lysed and subjected to western blotting as described above.

### Immunoprecipitation

Cells were lysed with IP Lysis/Wash Buffer (Thermo Scientific). The lysates were collected using a scraper and centrifuged at 13,000 RPM for 10 min and the supernatant was transferred to a new tube. The protein concentration of the cell extract was determined using a BCA Protein Assay Kit. Equal amounts of each protein sample (~500 μg/lane) were incubated with anti-SIRT1 and ubiquitin monoclonal antibodies (1:500) at 4° C, with overnight shaking. The immunocomplexes were incubated with Pierce Protein A/G Agarose for 1 h. After washing, the samples were precipitated with conditioning buffer, followed by immunoblot analysis.

### Preparation of nuclear and cytoplasmic extracts

After appropriate treatment, cells were washed twice with ice-cold PBS, collected using a scraper, and centrifuged at 3,000 RPM for 5 min at 4 °C. After removing the supernatant, 400 μL buffer A (1 mL 1.0 M HEPES (pH 7.9), 0.5 mL 2.0 M KCl, 0.02 mL 0.5 M EDTA (pH 8.0), 0.1 mL 0.1 M EGTA (pH 7.0), 96.28 mL sterile H_2_O) was added to the samples, vortexed gently, and then incubated on ice for 15 min. Thereafter, 25 μL 10% cNP-40 was added to the samples and vortexed strongly, followed by centrifugation at 13,000 RPM for 1~2 min at 4 °C. Supernatants were transferred to a new tube, and the above steps comprising treatment with buffer A and cNP-40 were repeated once more. The supernatants were used as the cytosolic extract, while 50 μL buffer B (1 mL 1.0 M HEPES (pH 7.9), 4.0 mL 5.0 M NaCl, 0.1 mL 0.5 M EDTA (pH 8.0), 0.5 mL 0.1 M EGTA (pH 7.0), 43.10 mL; sterile H_2_O) was added to the pellet fractions and vortexed strongly for 10 min. Thereafter, the cells were centrifuged at 13,000 RPM for 10 min at 4 °C; the supernatants were used as nuclear extracts.

### Immunoflourescence staining

The cells were fixed with 4% paraformaldehyde for 15 min at 23°C and washed thrice with PBS. Thereafter, cells were incubated with cold 4% paraformaldehyde for 10 min at -20 °C and washed for 5 min. After blocking with 1% BSA in PBS for 60 min, cells were treated with 0.4% Triton X-100 for 1 h. Thereafter, the cells were incubated with primary antibodies (rabbit anti-SIRT1 antibody [1:100 PBST] overnight at 4 °C, followed by incubation with the corresponding secondary antibodies (Alexa red anti-mouse IgG [1:500 diluted in PBST] for 1 h at 23 °C. After staining with DAPI (0.5 mg/mL), the cells were washed thrice with PBS, for 5min each, and observed using an EVOS fluorescence microscope (20×).

### 20S proteasome activity assay

Next, 200ul of the cell suspension (1.5 × 10^4^ cells/mL) was seeded in 96-well plates. Cells were treated with cisplatin or MG132 for 72 h. Thereafter, the medium was gently removed and replaced with 100µL of proteasome 20S activity assay solution, and the plate was analyzed and incubated for 24 h. The fluorescence was measured at an excitation wavelength of 380 nm and emission wavelength of 460 nm, using a spectrophotometer (Tecan Sunrise).

### Lung cancer patient DMFS (distant metastasis-free survival) analysis

SIRT1 expression affects patient survival: The association of SIRT1 expression with the survival rate of lung cancer patients was analyzed by Kaplan-Meier plotter (http://kmplot.com/analysis) for 1926 patients with affymetric probe 2178878_s_at. Patients were grouped as having high or low SIRT1, and median expression was used as the cutoff.

### Statistical analysis

All data are expressed as the mean standard deviation (SD). Significance between experimental and control groups was determined by t-test analyses. A probability (P) value < 0.05 was considered statistically significant.

## Results

### Cisplatin-resistant NSCLC cells exhibit mesenchymal-like cell characteristics and differences in SIRT1 mRNA levels

**Fig 1:**
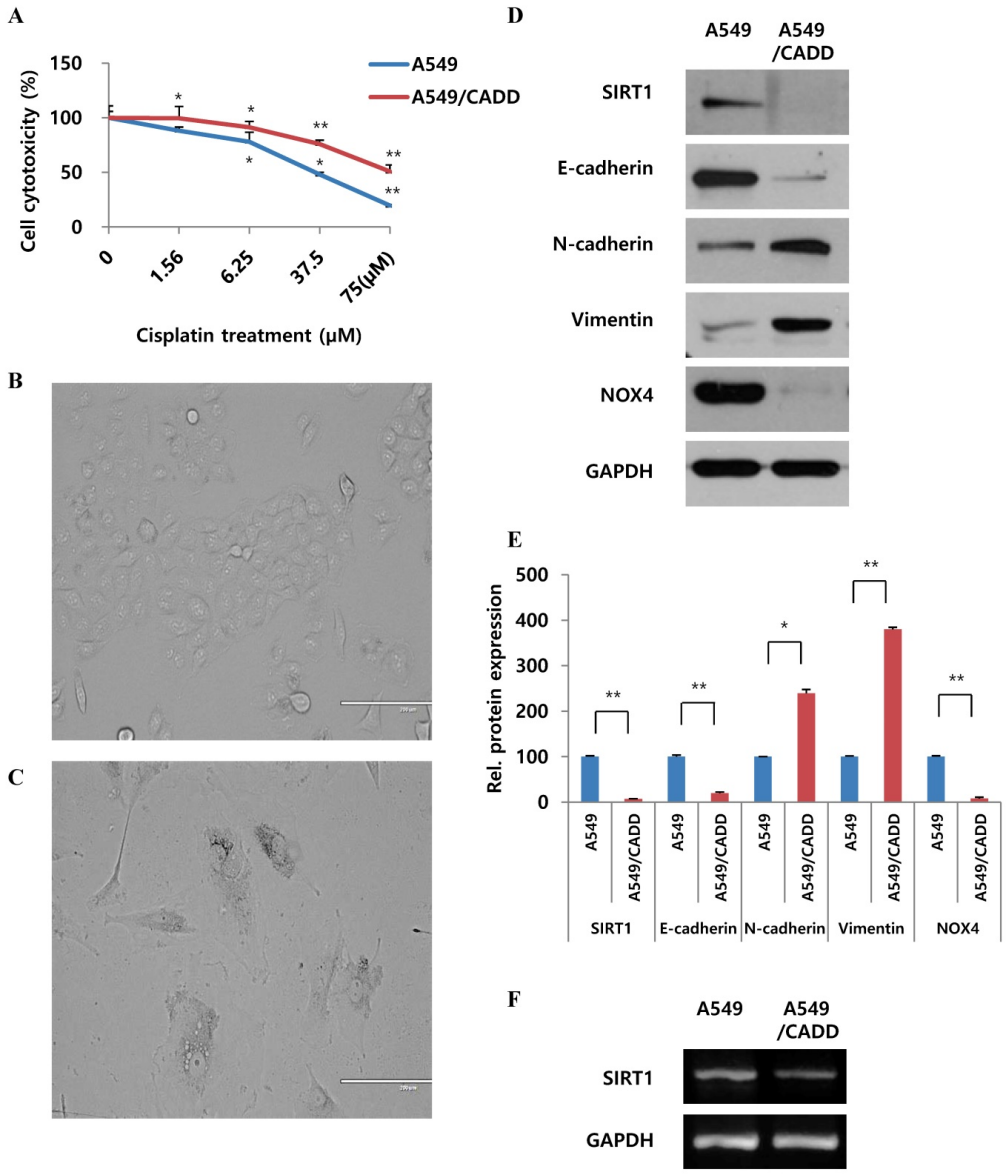
** Cisplatin-resistant NSCLC cells exhibit mesenchymal-like cell characteristics and differences in SIRT1 mRNA levels.** (A) MTT assay: A549 and cisplatin-resistant A549 cells (A549/CADD; 1.5 × 10^4^ cells/mL) were seeded in 96-well plates and treated with several cisplatin concentrations for 72h. (B) A549 phenotype as observed under a microscope. (C) A549/CADD phenotype as observed under a microscope. Scale bar, 200µm. (D) Western blotting to compare A549 cells and mesenchymal-like characteristics of A549/CADD cells. Expression of EMT markers was measured. (E) Quantification of signals normalized to GAPDH loading controls. *, p<0.05 and **, p<0.001 as compared to GAPDH. (F) RT-PCR for SIRT1 expression in A549 and A549/CADD cells.

In order to observe cell viability, cells were treated with 0-75 μM cisplatin. A significant increase in survival was observed, demonstrating a 2-fold increase between normal and A549/CADD cells (Fig. 1A). We repeatedly subculture in the presence of increasing concentration (0.1, 0.2, 0.4 and 0.5 mg/ml) of cisplatin over 6 months. Thereafter, the A549/CADD cell line was generated by colony selection, and maintained with 2 mg/L cisplatin over 2 months. Upon characterizing of cells after colony selection following cell exposure to cisplatin, a significant difference in the morphology between A549 cell lines (Fig. 1B) and their corresponding cisplatin-resistant sublines was observed, indicating the emergence of a mesenchymal morphology in the resistant sublines (Fig. 1C). Representative western blots revealed that the A549/CADD cells exhibited elevated expression of the mesenchymal markers N-cadherin and vimentin and depleted levels of SIRT1 and epithelial marker E-cadherin, in parallel with inducing EMT (Fig. 1D); GAPDH was used as a background control (Fig. 1E). As drug resistance is associated with hypoxic condition, we looked for hypoxic related factors such as NAPDH oxidase (NOX). Interestingly, the chemo resistant cells showed NOX4 downregulation. In addition, representative RT-PCR results indicated that A549/CADD cells showed SIRT1 downregulation (Fig. 1F).

### Cisplatin changes signaling and inhibits proliferation of cisplatin-resistant NSCLC cells and induces G1 arrest

**Fig 2:**
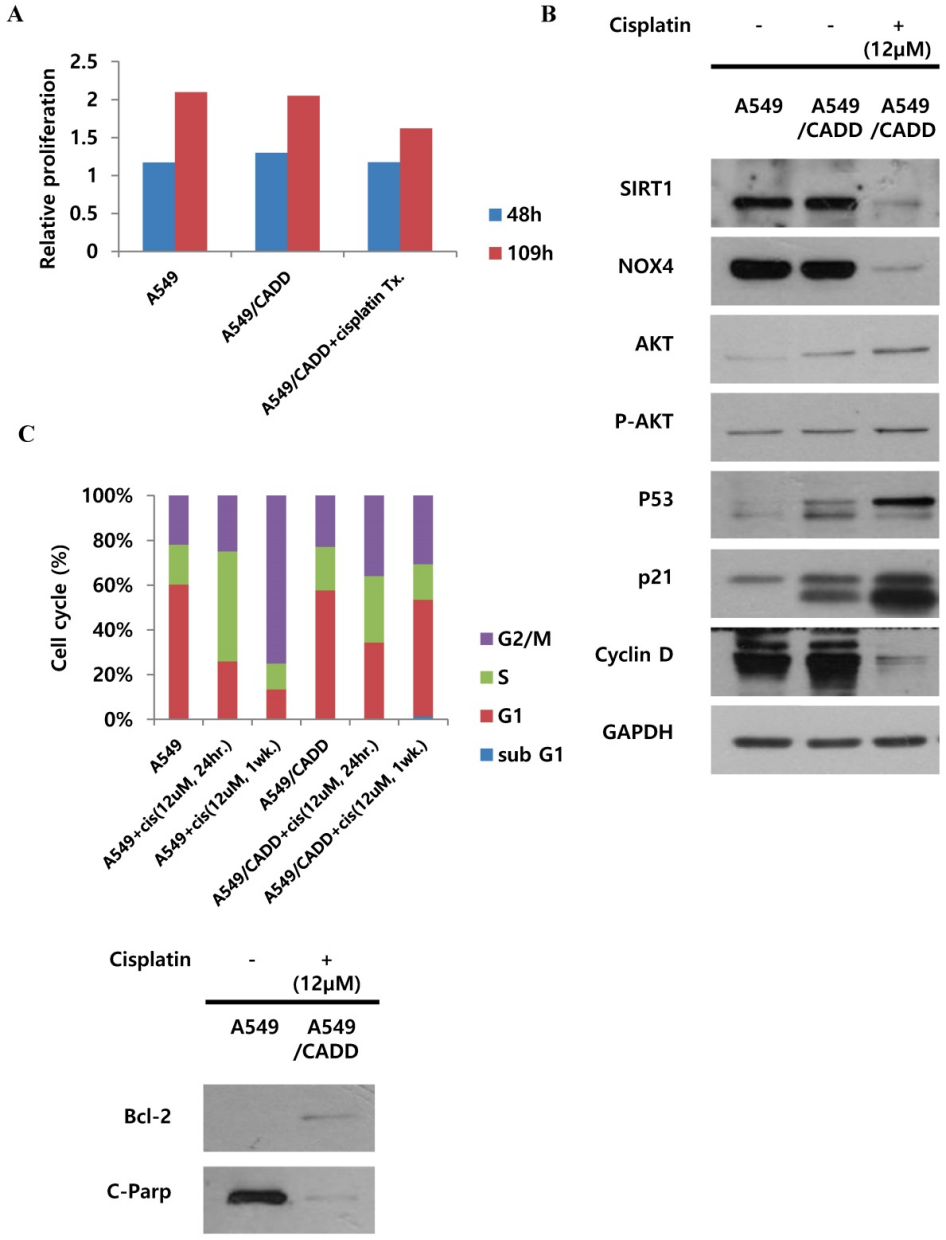
** Cisplatin changes signaling and inhibits proliferation of cisplatin-resistant NSCLC cells and induces G1 arrest.** (A) Detection of real-time proliferation of A549 cells, adaptive A549/CADD cells, and A549/CADD cells (3 × 10^3^ cells/mL) seeded in 8- well plates pre-coated with 10 mM L-cysteine (electrode-stabilizing solution). A549/CADD cells were treated with 12 μM cisplatin for 25-109 h using an ECIS machine. The resistance was calculated as a ratio compared to 0 h. (B) Western blot signals of adaptive A549/CADD cells were reversed and A549/CADD cells were retreated with cisplatin for 1week. Western blotting for detection of SIRT1, NOX4, cell cycle factors, p53 and p21, and pro-survival factor, AKT in A549 and A549/CADD cells. (C) Flow cytometry for determining the distribution of A549 cells and 12µM cisplatin treated (for 24 h and 1week) A549/CADD cells in various phases of the cell cycle phases. Western blotting for detection of anti-apoptosis signaling molecule, Bcl-2, and apoptotic factors, cleaved PARP in A549 and A549/CADD cells.

We demonstrated the proliferative capacity of A549, A549/CADD, and cisplatin-treated A549/CADD cells (treated with 12 μM cisplatin from 25 h after cisplatin exposure); at 109 h, proliferative capacity of the cells was found to be significantly decreased in the A549/CADD cells that were treated with cisplatin for 1 week. (Fig. 2A). We were intrigued whether the cell cycle factors are involved in slowing the cell growth rate. It is known that SIRT1 modulates p53-mediated growth regulation 9. Moreover, among the cell cycle factors, p53 can arrest growth by holding the cell cycle at the G1/S regulation point in the DNA damage process. In addition, p21, also known as cyclin-dependent kinase inhibitor 1, represents a major target of p53 activity and is therefore associated with DNA damage to cell cycle arrest 13. In conclusion, we observed that cisplatin treatment induced changes in several signaling molecules in the A549/CADD cells; firstly, p53, p21, pro-survival factor AKT, and anti-apoptosis signaling molecule Bcl-2, were upregulated, while levels of SIRT1, NOX4 and apoptotic factors such as cleaved PARP were downregulated. (Fig. 2B, C). Our findings suggest that cisplatin treatment induced anti-apoptosis and cell cycle alterations in A549/CADD cells. Anti-apoptosis factor Bcl-2 and cell cycle factors p53 and p21 were upregulated, while cyclin D was downregulated presumably because cyclin D drives the G1/S phase transition. We assumed that cell cycle factors such as p53, p21, and cyclin D changed the cell proliferative capacity. We further confirmed the distribution of the cells in the various cell cycle phases in response to cisplatin treatment. After 12 μM cisplatin treatment for 1 week, an increased accumulation of G1-phase cells was observed in A549/CADD cells relative to the A549 cell line (Fig. 2C). These alterations in cell distribution in the various cell cycle phase may play an important role in the cisplatin-resistant phenotype of the generated NSCLC cell lines. Thus, when cisplatin treatment of the A549/CADD cells was discontinued after 1 or 2 weeks, the observed difference between A549 and A549/CADD cells were alleviated and the A549/CADD cells regained the characteristic features of the original A549 cell line. The expression of SIRT1 was increased again, and cell morphology was gradually changed to A549 cells. To overcome this problem, we treated the A549/CADD cells with cisplatin for 1week, before performing the experiment and compared the difference in cell cycle factors and signaling mediators between A549, A549/CADD (cisplatin treatment discontinued) and A549/CADD (with appropriate amount of cisplatin treatment for a week before the experimental observation). From now on, the cisplatin treated A549/CADD cells were taken into consideration for further studies and were denoted as A549/CADD cells.

### Unlike cisplatin-resistant NSCLC cells, reduced SIRT1 induced anti-apoptosis and cell cycle arrest through p53 and p21 in lung cancer cells

**Fig 3:**
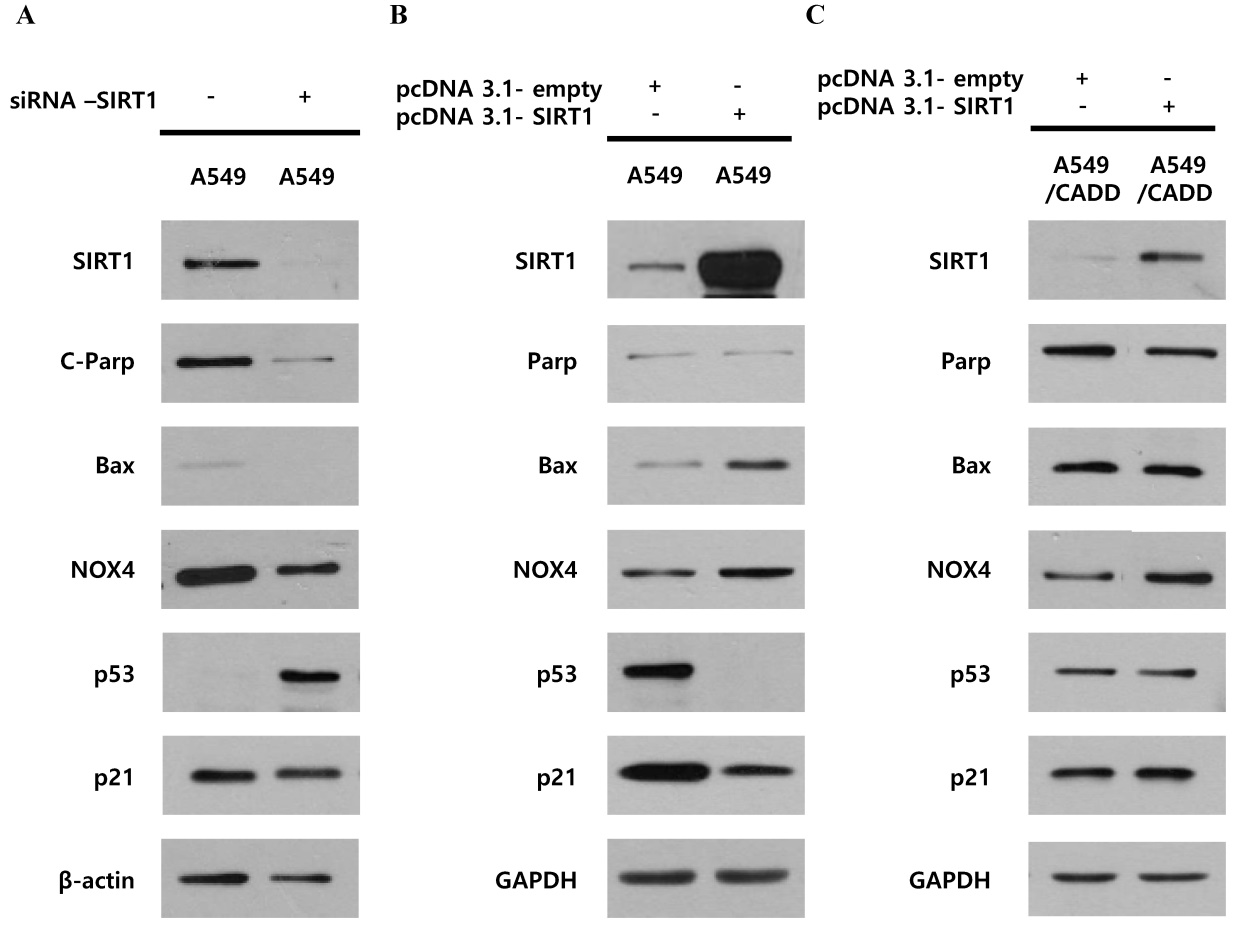
** Unlike cisplatin-resistant NSCLC cells, reduced SIRT1 induced anti-apoptosis and cell cycle arrest through p53 and p21 in lung cancer cells.** (A) Western blotting for studying the SIRT1 knockdown effect of NOX4, cell cycle factors, p53 and p21, and apoptosis signals Bax and cleaved PARP in A549 cells. (B) Western blotting for studying SIRT1 overexpression effect on NOX4, cell cycle factors, p53 and p21, and apoptosis signals, Bax and total PARP in A549 cells. (C) Western blotting for studying the SIRT1 overexpression effect of NOX4, cell cycle factors, p53 and p21, and apoptosis signals Bax and total PARP in A549/CADD cells.

We investigated whether decreased SIRT1 expression induced p53, p21 and cell cycle changes in A549/CADD cells by studying the effect of knockdown or overexpression of SIRT1. Firstly, SIRT1 knockdown reduced apoptosis markers, c-PARP, Bax, and NOX4 and increased cell cycle factor p53 in A549 cells (Fig. 3A). SIRT1 overexpression reduced cell cycle factors, p53 and p21 in A549 cells (Fig. 3B). These results imply that SIRT1 downregulation induced anti-apoptosis and cell cycle inhibition through p53 and p21 upregulation in A549 cells. However, SIRT1 overexpression in A549/CADD cells was not dramatic and had little effect (Fig. 3C). Therefore, we aimed to investigate the mechanism that inhibits SIRT1 expression or the induction of p53 degradation in A549/CADD cells.

### Cisplatin treated cisplatin-resistant NSCLC cells induced p53 stability and inhibited p53 ubiquitination and SIRT1 expression

**Fig 4:**
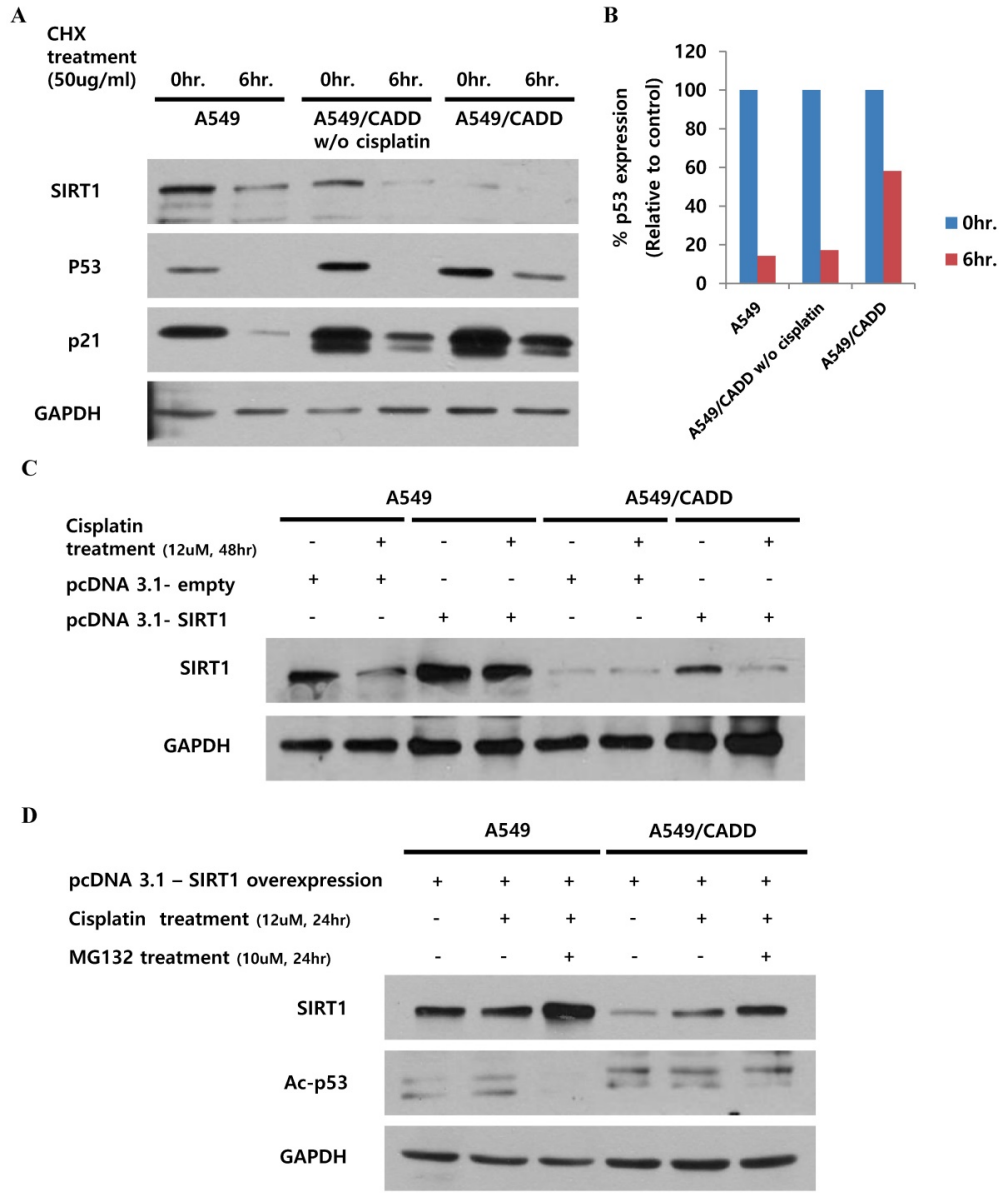
** Cisplatin treatment of cisplatin-resistant NSCLC cells induced p53 stability and inhibited p53 ubiquitination and SIRT1 expression.** (A) Western blotting to examine the effect of 12uM cisplatin on protein stability (in the absence of new protein synthesis) by adding 50 ng/mL cycloheximide (CHX) for 6 h. (B) Quantification of p53 signals normalized to GAPDH loading controls. *, p<0.05 and **, p<0.001 as compared to A549 cells. (C) Western blotting to examine the effect of SIRT1 overexpression when cells were treated with 12 µM cisplatin for 48 h in A549 and A549/CADD cells. (D) Western blotting to examine the SIRT1 overexpression effect and acetyl-p53 levels by adding 12 µM cisplatin for 24 h and 10 µM proteasome inhibitor MG132 for 24 h.

Our finding indicated that the observed change in p53, subsequent to SIRT1 depletion, may have occurred by altering p53 stability in A549/CADD cells. Recent studies have demonstrated the site structure of p53 and its relation to chemoresistance 14, and the acetylation site and ubiquitination sites are competitive 15. We speculated that the induction of p53 acetylation by depleted SIRT1 might mediate the reduction of p53 ubiquitination in A549/CADD cells. Therefore, we investigated cell cycle factors and p53 stability in A549/CADD cells. Accordingly, we treated A549 and A549/CADD cells (with or without cisplatin treatment) with the protein synthesis inhibitor cycloheximide and collected the cells at 0 and 6h post-treatment (Fig. 4A, B). Compared to A549 cells, cycloheximide decreased p53 expression in cisplatin-treated A549/CADD cells. These data indicate that the SIRT1 expression level induced p53 stability, because depleted SIRT1 decreased the effect of cycloheximide through decreased p53 ubiquitination in cisplatin-treated A549/CADD cells. We further wondered whether cisplatin affected post-translational modification or ubiquitination of SIRT1. A recent finding indicates that cell survival and death are related to SIRT1 ubiquitination 16. Therefore, we examined SIRT1 ubiquitination by transfecting A549/CADD cells with SIRT1 for 24 h, followed by cisplatin treatment for 48 h (Fig. 4C). Our results suggest that SIRT1 expression was suppressed by cisplatin in A549/CADD cells, transfected with SIRT1. Further, to determine whether cisplatin modifies proteasome activity, we first treated the A549/CADD cells (transfected with SIRT1 vector) with 10 µM proteasome inhibitor MG132 for 24 h. As shown in Fig. 4D, exogenous SIRT1 was upregulated and acetyl-p53 was downregulated in MG132-treated A549/CADD cells, because depleted SIRT1 influenced p53 acetylation. This finding implies that cisplatin treatment significantly decreased SIRT1 protein levels and increased proteasomal activity.

### Cisplatin affects SIRT1 ubiquitination in the cytoplasm of cisplatin-resistant NSCLC cells

**Fig 5:**
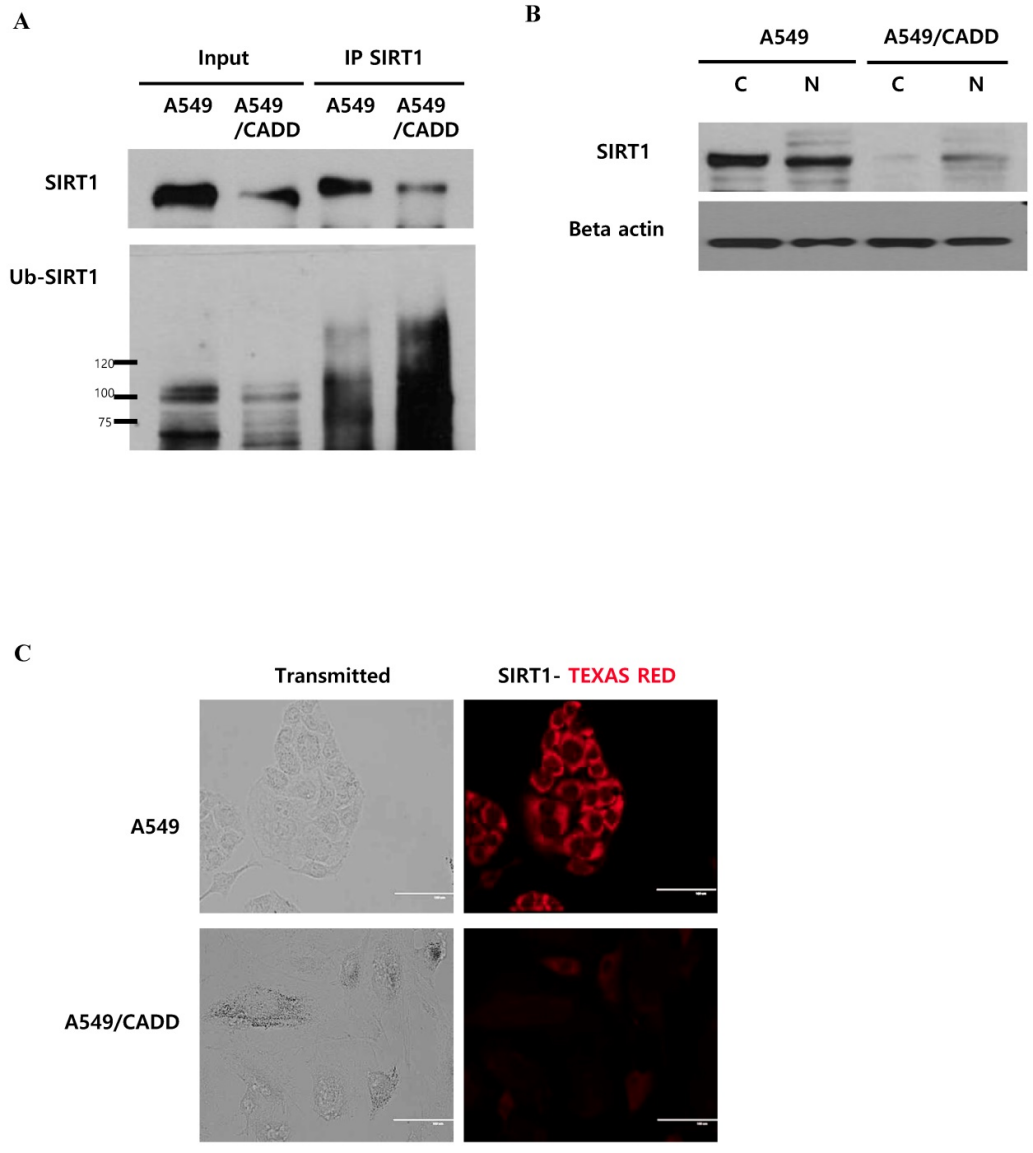
** Cisplatin affects SIRT1 ubiquitination in the cytoplasm of cisplatin-resistant NSCLC cells.** (A) Immunoprecipitation to examine SIRT1 ubiquitination in A549 and A549/CADD cells using anti-SIRT1 and ubiquitin antibodies. (B) A549 and A549/CADD cells were treated with 12µM cisplatin for 1week, followed by the preparation of nuclear (N) and cytoplasmic (C) extracts for western blot analysis using SIRT1 antibody. (C) SIRT1 was immunostained with anti-SIRT1 antibody (1:500 dilution) and Alexa red anti-mouse IgG (1:500 dilution in PBST) in A549 and A549/CADD cells. Scale bar, 100µm.

As shown in Figure 4, we demonstrated the downregulation of SIRT1 level and induction of proteasomal activity in A549/CADD cells. Next, we conducted SIRT1 immunoprecipitation studies in order to investigate whether SIRT1 ubiquitinaton levels were altered in A549/CADD cells. We observed that endogenous SIRT1 immunoprecipitated with SIRT1 antibody. Further, we assessed the levels of SIRT1 ubiquitination using an ubiquitin antibody in the SIRT1 immunoprecipitate (Fig. 5A). Indeed, the SIRT1 ubiquitination level was increased in A549/CADD cells. Several studies have shown that nucleocytoplasmic shuttling of SIRT1 may play a role in inhibiting cell death and several other cell functions. Therefore, we performed a fractionation experiment to investigate the site of SIRT1 degradation and to reveal the relationship between degraded SIRT1 localization and resistant mechanisms, such as cell cycle arrest and apoptosis, in A549/CADD cells. Following fractionation, each fraction was loaded with the same volume ratio in A549 and A549/CADD cells. Consequently, we confirmed that SIRT1 was degraded in the cytoplasm of A549/CADD cells (Fig. 5B). While SIRT1 was originally found as a nuclear protein, recent reports have shown that SIRT1 is not anchored only in the nucleus 17; rather, SIRT1 dynamically shuttles between the cytoplasm and nucleus 18. We found that SIRT1 was partially localized in the cytoplasm in A549/CADD cells. In A549 cells, endogenous SIRT1 was mainly localized in the cytoplasm, while partial nuclear localization was also observed. However, endogenous SIRT1 was mainly localized in the nuclei in A549/CADD cells and was almost absent in the cytoplasm. Recent reports have shown that cells with cytoplasm-localized SIRT1, exhibit enhanced apoptosis 19. Therefore, we anticipate that the alteration in sub-cellular SIRT1 localization is orchestrated in the cytoplasm by cisplatin treatment. We further confirmed sub-cellular SIRT1 localization by immunofluorescence; endogenous SIRT1 staining was found to be very weak in the A549 nuclei. In contrast, the remaining SIRT1 was observed only around and in the nuclei of A549/CADD cells (Fig. 5C). These data suggested that cisplatin affected only cytoplasmic SIRT1 through SIRT1 ubiquitination and degradation.

### Cisplatin regulates SIRT1 degradation via proteasomal activity in cisplatin-resistant NSCLC cells

**Fig 6:**
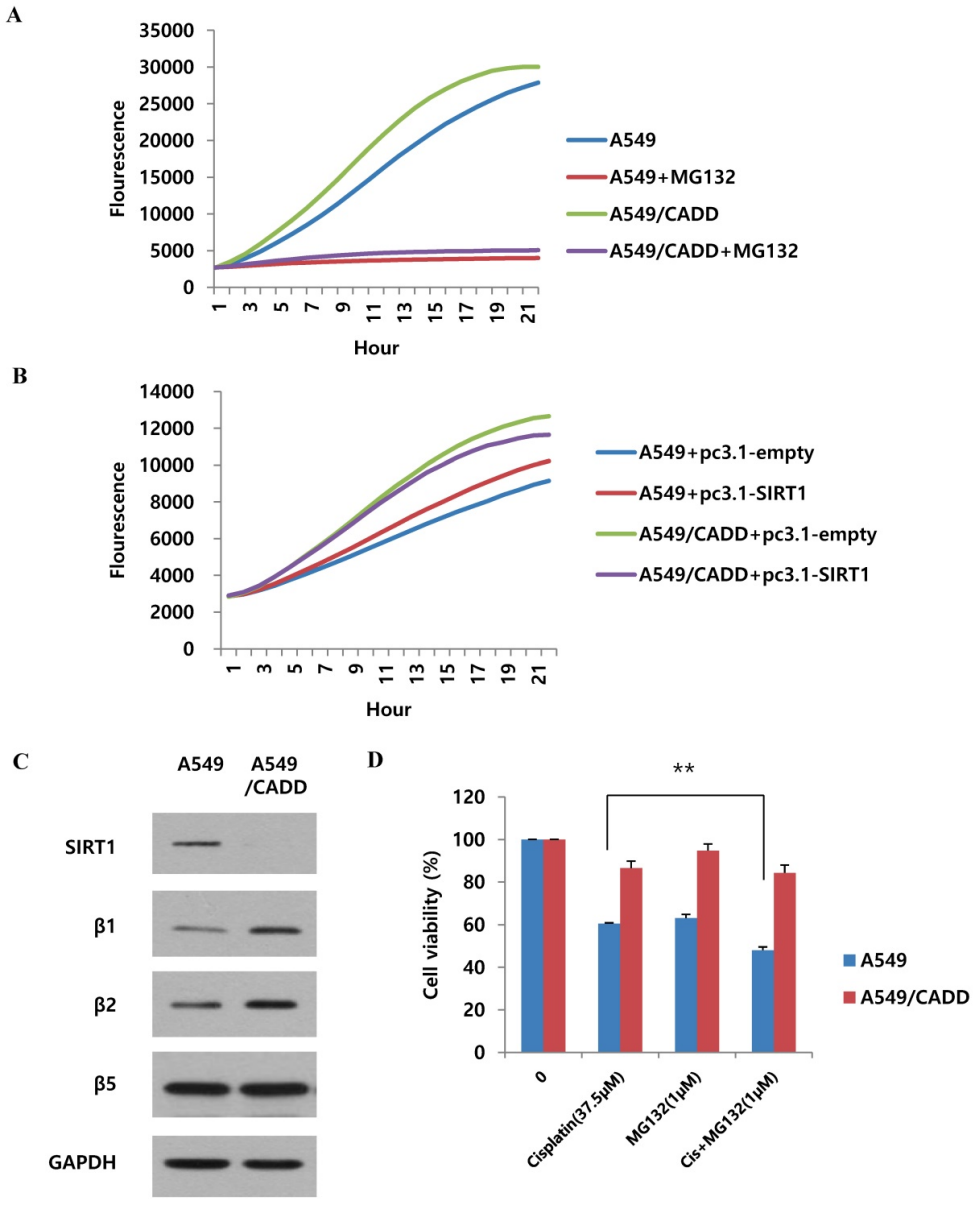
** Cisplatin regulates SIRT1 degradation via proteasomal activity in cisplatin-resistant NSCLC cells.** (A) 20S proteasome activity assay to examine the effect of cisplatin in A549 and A549/CADD cells. We confirmed that 5µM MG132 treatment reduced proteasomal activity in both A549 and A549/CADD cells. (B) 20S proteasome activity assay for determine the overexpression efficacy of SIRT1 in A549/CADD cells. (C) Western blotting to study the effect of cisplatin on proteasome subunits in A549/CADD cells. (D) MTT assay of A549 and A549/CADD cells treated with 37.5 µM cisplatin and 1 µM MG132 for 24 h.

As shown in Fig. 5, we found that SIRT1 ubiquitination increased in A549/CADD cells. Therefore, we wondered whether proteasomal activity also increased in A549/CADD cells. Recent studies have shown that cisplatin is associated with proteasomal activity 20. Therefore, we measured the 20S proteasome activity in A549 and A549/CADD cells. Indeed, compared to A549 cells, the proteasomal activity of A549/CADD cells was increased (Fig. 6A). The relative activities indicated that cisplatin induced upregulated 20S proteasome activity. Thereafter, we investigated the effect of SIRT1 overexpression in A549 and A549/CADD cells and found that A549/CADD cells showed greater proteasomal activity than A549 cells, which was the same tendency as that observed in the absence of overexpression (Fig. 6B). Therefore, the increase in proteasomal activity in cisplatin-treated A549/CADD cells was not due to the SIRT1 decrease. Rather, the decrease in SIRT1 in A549/CADD cells was due to increased proteasomal activity caused by cisplatin. Moreover, we found that A549/CADD cells showed greater proteasomal activity than A549 cells in Fig. 6B. Therefore, we demonstrated that the proteasome activation mechanism made the SIRT1 overexpression invisible to significant changes in Fig. 3C. We further investigated which proteasome subunit was affected by cisplatin in A549/CADD cells. We observed that the β1 and β2 subunits were increased by cisplatin (Fig. 6C). Therefore, our results suggest that cisplatin increased the expression of proteasome subunits β1 and β2, thereby increasing proteasomal activity in A549/CADD cells. Further, we aimed to investigate the effect of proteasome inhibitor MG132 and its synergy with cisplatin. We treated A549 and A549/CADD cells with 37.5 µM cisplatin (Fig. 1C.) and 1 µM MG132 for 48 h and found significantly increased cell cytotoxicity in co-treated A549 cells (Fig. 6D). However, this effect was not seen in A549/CADD cells. This implies that proteasome inhibitor MG132 showed a synergistic effect with cisplatin in A549 cells. Further studies are needed to understand the effect of proteasome inhibition in A549/CADD cells. Together, our results reveal a proteasome activation mechanism, driven especially by the upregulation of the β1 and β2 subunits in A549/CADD cells.

### Adriamycin regulates proteasomal activity and other factors in adriamycin-resistant A549 cells

**Fig 7:**
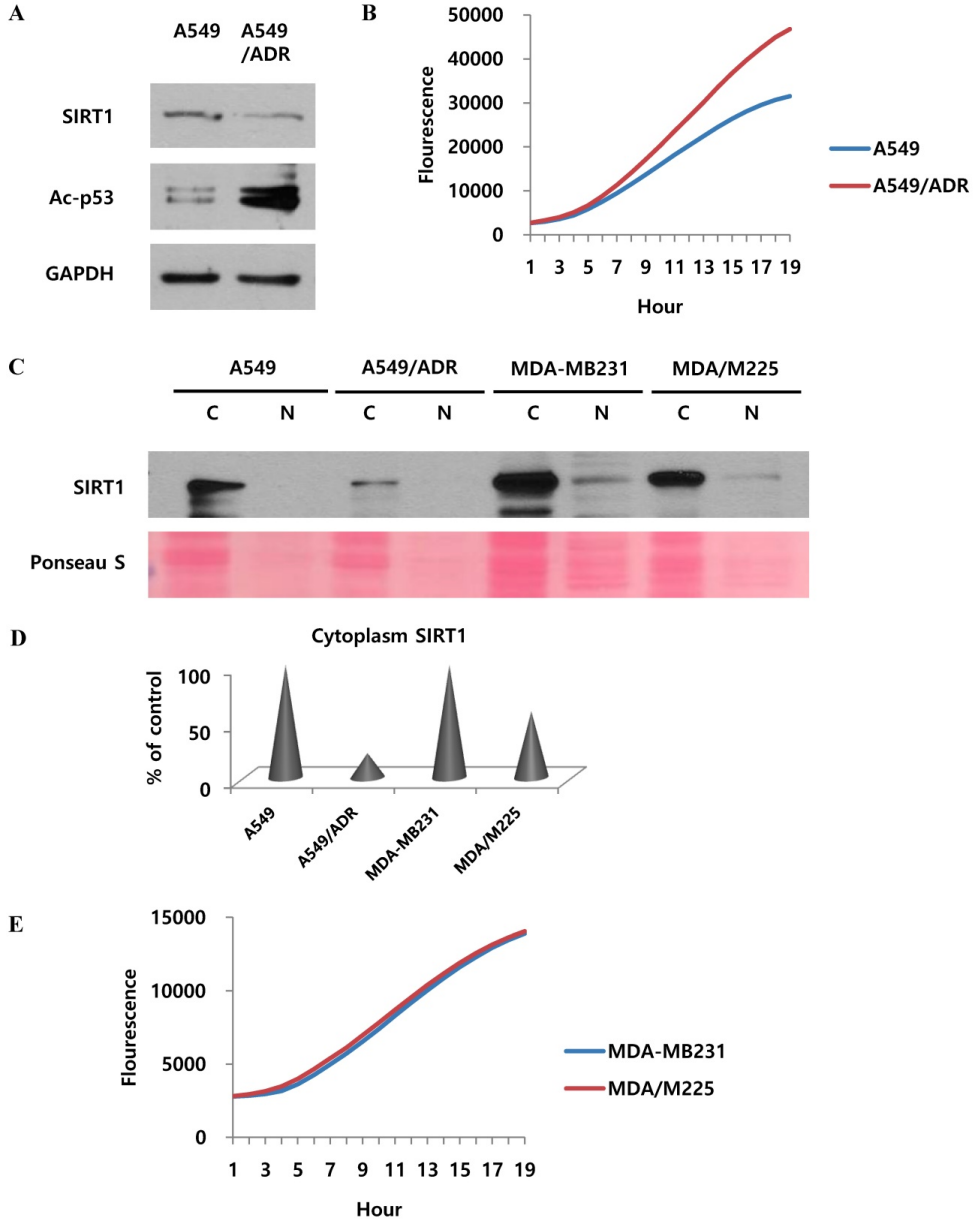
** Adriamycin regulates SIRT1 proteasome activity and other factors in adriamycin-resistant A549 cells.** (A) Western blotting to study the effect of adriamycin on SIRT1 and acetyl-p53 levels in A549 cells and adriamycin-resistant A549 cells. (B) 20S proteasome activity assay to determine the effect of adriamycin on A549 and A549/ADR cells. (C) Western blotting to detect the SIRT1 level in nuclear (N) and cytoplasmic (C) fractions of A549, A549/ADR, MDA-MB231, and 12Gy radiation resistant (MDA/M225) cells. (D) Quantification of cytoplasmic SIRT1, normalized to control cells. (E) 20S proteasome activity assay to study the effect of radiation on MDA/M225 cells.

We wondered if the results of our study also apply to the mechanism of adriamycin-resistance in A549 cells. Indeed, the effect of cisplatin was demonstrated in adriamycin-resistant A549 cells as well as A549/CADD cells. Our results revealed that SIRT1 was downregulated and acetyl-p53 was upregulated in both A549/ADR cells and A549 cells (Fig. 7A). We further investigated the 20S proteasome activity in A549 and A549/ADR cells and found increased proteasomal activity in the latter (Fig. 7B). The relative activities indicated that adriamycin induced upregulated 20S proteasome activity. This implies that adriamycin exerted the same effect as cisplatin, in resistant A549 cells. Thereafter, we investigated each fraction of other resistant A549 cells, including adriamycin-resistant A549 and 12 Gy radiation-resistant MDA-MB231 cells (Fig. 7C). We demonstrated that SIRT1 was degraded in the cytoplasm of A549/ADR cells as well as A549/CADD cells. This result was not observed significantly in MDA-MB231/M225 cells. Therefore, we normalized the cytoplasmic SIRT1 level of ADR- and M225-resistant cells to that of the control cells (Fig. 7D). We further examined the 20S proteasome activity in MDA-MB231 and MDA-MB231/M225 cells and found that the proteasome activity was not significantly different among the two cells (Fig. 7E). Our data suggested that adriamycin has the same mechanism of cytoplasmic SIRT1 degradation as that of cisplatin, in resistant A549 cells. We excluded the possibility of resistant mechanisms of other radiation-induced resistant cells.

## Discussion

EMT is implicated in cancer progression and is considered as the main regulator in radio-chemo resistance, a major hurdle in cancer treatment 8. The divergent role of sirtuin, an NAD^+^ dependent acetylase, in cancer progression, and its association with EMT is poorly understood 5. Furthermore, nucleoplasmic shuttling of SIRT1 varies in different tissues and in *in -vivo* models, which offers an interesting view on their regulation of cell cycle mechanisms, particularly in cisplatin-resistant cells 12. In the current study, we found that cisplatin influences cell cycle arrest and affects p53 acetylation in A549/CADD cells. We also found that upon cisplatin treatment, cytoplasmic degradation of SIRT1 is observed. Furthermore, cisplatin was found to induce total and activated AKT expression as well as diminish NOX4 expression in A549/CADD cells.

In order to investigate the existence of possible connections between SIRT1 and p53 in cisplatin-resistant cells, we upregulated/downregulated SIRT1 expression and anlayzed its impact on cell cycle events and apoptosis. While NOX4 and Bax expression was found to be higher in SIRT1-overexpressed A549 cells, the expression of cell cycle inhibitors such as p53, p21, and PARP was decreased. This result reverses upon SIRT1 inactivation. Further, SIRT1-overexpressing A549/CADD cells treated with cisplatin showed induced actyl-p53 possibly via inhibiting histone deacetylases. The enhanced p53 acetylation might result in ac-p53-dependent activation of apoptosis in A549/CADD cells, but not in A549 cells. In addition, SIRT1 availability in cisplatin-treated A549/CADD cells was partially decreased owing to cisplatin-induced proteasomal activity 21.

In general, the induction of SIRT1-ubiquitination followed by proteasome-mediated SIRT1 degradation reduces its protein level, thereby participating in the pathological development of cell senescence. Further, inhibition of proteasomal activity enhances the cisplatin sensitivity of cancer cells in osteosarcoma 22. Therefore, we envisage an inhibitory mechanism of SIRT1 in cisplatin-resistant NSCLC. Moreover, our experimental immunoprecipitation data showed that cisplatin induces SIRT1 ubiquitination in A549/CADD cells. Next, we measured the 20S proteasomal activity in A549 and A549/CADD cells and found elevated proteasomal activity in A549/CADD cells. Cisplatin treatment induced the expression of proteasome subunits such as β1 and β2 in A549/CADD cells.

The role of SIRT1 in cancer cell death and progression is controversial because SIRT1 has both tumor-promoting 11 and tumor-suppressing functions 23. Therefore, we investigated SIRT1 regulation in other resistant cell lines, including adriamycin-resistant A549 and radiation-resistant MDA/MB231 cell lines. Interestingly, we found acetyl-p53 expression in A549/ADR, but not in 12Gy radiation-resistant MDA/MB231 cells. Generally, SIRT1 is expressed in all cell types and largely identified as a nuclear protein, with sparse presence in the cytoplasm in certain cancer cell lines, such as A549 cells 12. Herein, we found that the SIRT1 cytoplasmic degradation mechanism was common to adriamycin-resistant NSCLC cell lines, but not to the radiation-resistant cells. Relatively reduced expression of cytoplasmic SIRT1 in A549/ADR cells compared to that in A549 cells induces anti-apoptosis and is associated with drug resistance, with increased proteasomal activity in cisplatin-resistant cells. In summary, cisplatin resistance increases proteasomal activity and cytoplasmic SIRT1 degradation. In addition, the cytoplasmic localization of SIRT1 induces cell cycle arrest and proliferation, while apoptosis is suppressed in cisplatin-resistant cells.

**Fig 8:**
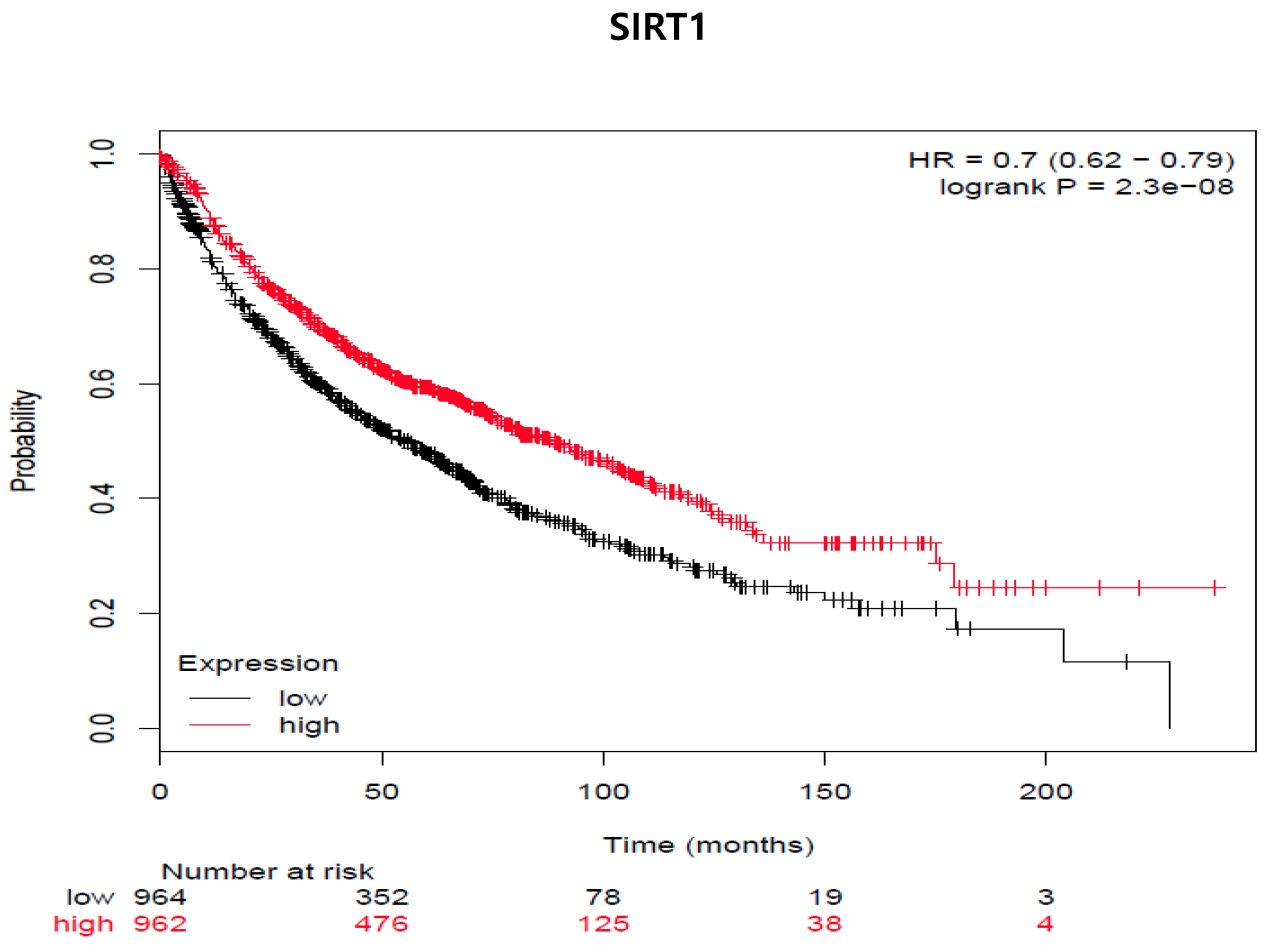
** SIRT1 expression is associated with decreased distant metastasis-free survival (DMFS) in all cancer patients (adenocarcinoma and squamous cell carcinoma cancer patients [n=1926]). The mRNA gene chip data was used for Kaplan Meier plotter analysis.** Patients were grouped as having 'high' (red) or 'low' (Black) SIRT1 expression, and median expression was used as a cutoff. HR = 0-7 (0.62-0.79), log-rank p-value = 2.3e-8.

So far, in preclinical studies, the therapeutic use of proteasome inhibitors is well documented during chemotherapy treatment 24. Therefore, we examined whether SIRT1 expression was associated with the survival rate of lung cancer patients (Fig 8). We used the program because we had not yet experimented samples of clinical patients. We analyzed the two groups through the Kaplan-Meier plotter program about lung cancer to see the impact of SIRT1 expression on relapse-free survival. We examined the free survival rate in association with SIRT1 expression by Kaplan-Meier plotter (http://kmplot.com/analysis) 25. Indeed, lower SIRT1-expressing patients showed curves that were associated with poor prognosis and lower relapse-free survival compared to patients with higher expression (n = 1926, Log-rank p-value = 2.3e-08, HR = 0.78, probe id: 218878_at).

In summary, SIRT1 induces pro-apoptotic activity of the acetylated transcription factor p53 in A549 cells, and cytoplasm-localized SIRT1 is related to cell cycle arrest in A549/CADD cells. Cytoplasm-localized SIRT1 overexpression represents a novel therapeutic target to inhibit drug resistance. We speculate that the activity of proteasome subunits β1 and β2 induces cisplatin resistance. Therefore, an inhibitor targeting proteasome subunits could be a new treatment strategy for patients with cisplatin-resistant NSCLC cells. Altogether, our findings address cisplatin-induced cell cycle arrest pathways, in which SIRT1 interacts with p53 and regulates proteasomal degradation through acetylation and the extended mechanism by which cisplatin regulates cell cycle arrest in A549/CADD cells. Further studies on the SIRT1-mediated DNA damage associated with cisplatin and their metabolic regulation in the EMT process are encouraged.

## Abbreviations

A549/CADD: cisplatin-resistant A549
CDDP: *cis*-diamminedichloroplatinum(II)
NSCLC: non-small cell lung cancer
EMT: Epithelial-mesenchymal transition
SIRT1: Sirtuin-1
NOX: Nicotinamide adenine dinucleotide phosphate oxidase
DMFS: distant metastasis-free survival
